# The Role of Plant Hormones in the Interaction of *Colletotrichum* Species with Their Host Plants

**DOI:** 10.3390/ijms222212454

**Published:** 2021-11-18

**Authors:** Thomas Svoboda, Michael R. Thon, Joseph Strauss

**Affiliations:** 1 Department of Applied Genetics and Cell Biology (DAGZ), Institute of Microbial Genetics, University of Natural Resources and Life Sciences, Vienna (BOKU), 3430 Tulln an der Donau, Austria; joseph.strauss@boku.ac.at; 2 Institute for Agribiotechnology Research (CIALE), Universidad de Salamanca, 37185 Salamanca, Spain; mthon@usal.es

**Keywords:** *Colletotrichum* spp., plant hormones, ethylene, auxin, jasmonic acid, salicylic acid, abscisic acid

## Abstract

*Colletotrichum* is a plant pathogenic fungus which is able to infect virtually every economically important plant species. Up to now no common infection mechanism has been identified comparing different plant and *Colletotrichum* species. Plant hormones play a crucial role in plant-pathogen interactions regardless whether they are symbiotic or pathogenic. In this review we analyze the role of ethylene, abscisic acid, jasmonic acid, auxin and salicylic acid during *Colletotrichum* infections. Different *Colletotrichum* strains are capable of auxin production and this might contribute to virulence. In this review the role of different plant hormones in plant—*Colletotrichum* interactions will be discussed and thereby auxin biosynthetic pathways in *Colletotrichum* spp. will be proposed.

## 1. Introduction

*Colletotrichum* spp. are opportunistic plant pathogenic fungi which are able to infect virtually every economically important plant species, causing diseases commonly referred to as anthracnose. Both monocotyledonous plants such as sorghum or maize as well as dicotyledonous plants such as strawberries, soya or stone fruits can serve as host plants [1,2,3,4,5]. Among the genus *Colletotrichum* different subspecies differ in their way of colonization as well as in obtaining nutrients. Their lifestyles range from biotrophs to hemibiotrophs to necrotrophs. Hemibiotrophic infection begins with a biotrophic phase before the pathogen switches to the necrotrophic phase, and they are therefore able to exist in the plant as endophytes [6,7,8]. Within the genus *Colletotrichum*, around 190 species are currently described which are organized into 11 species complexes and 23 singleton species [9].

For a successful infection, *Colletotrichum* forms appressoria to penetrate the host plant. Around 4000 genes have been found to be upregulated in the appressoria of *C. gloeosporioides* growing on PDA covered with cellophane and among those, 468 genes are exclusively expressed in the appressoria and not in hyphal tissue [10]. These upregulated genes can be assigned to 107 KEGG pathways including secondary metabolism and molecular transport [10]. Analysis of the transcriptome of *C. fructicola* revealed 3189 genes differentially expressed in four infection-related structures (conidia, appressoria, infected apple leaves, cellophane infectious hyphae). Among the upregulated genes, small secreted proteins, cytochrome P450 mono-oxygenases, carbohydrate-active enzymes (CAzymes) and secondary metabolite synthetases were enriched [11]. Genes encoding CAzymes potentially degrade the plant cell wall and are more abundant in the genomes of hemibiotrophic and necrotrophic pathogens than in biotrophs [12]. 

Rho GTPases play a crucial role in signal transduction regulating morphogenesis and differentiation. In *C. gloeosporioides*, disruption of CgCdc42 results in reduced formation of appressoria which are morphologically abnormal. Furthermore, ΔCgCdc42 mutants exhibit hypersensitivity towards H_2_O_2_ and transcriptional analysis suggesting that the gene plays a role in the regulation of ROS-related genes [13]. In *C. obiculare*, the causal agent of cucumber anthracnose, fatty acid β-oxidation in peroxisomes is critical for the appressorial melanisation and lipolysis [14].

The main phytohormones produced upon biotic and abiotic stresses are abscisic acid (ABA), salicylic acid (SA), jasmonic acid (JA) and ethylene (ET) [15,16]. Increasing levels of JA, SA and ET upon infection indicate that these hormones primarily mediate the response upon biotic stresses [15]. On the other side ABA biosynthesis is enhanced when abiotic stresses like heat, drought, salinity or cold prevail [17,18]. Because of different interactions between hormones the stress response is not only restricted to JA, SA, ET and ABA. Auxins (IAA), gibberellins (GA) and cytokines (CK) have also been reported to play a role in the regulation of the plant defense response [15,19,20]. Comparative transcriptomic analysis of maize infected with *C. graminicola* revealed an accumulation of SA inducible genes as well as accumulation of transcripts involved in JA and ET signaling pathways suggesting a hormone signaling crosstalk in systemic female inflorescence inoculated with *C. graminicola* [21]. There are also other transcriptomic studies available where the response of different plants like *Phaseolus vulgaris* [22], octoploid strawberry [23] or postharvest mango fruits [24] upon *Colletotrichum* infection were analysed. In this review, the role of JA, SA, ET, IAA, ABA and GA in plant-pathogen interaction of different *Colletotrichum* spp. affecting various hosts will be discussed.

## 2. Ethylene

**Figure 1:**
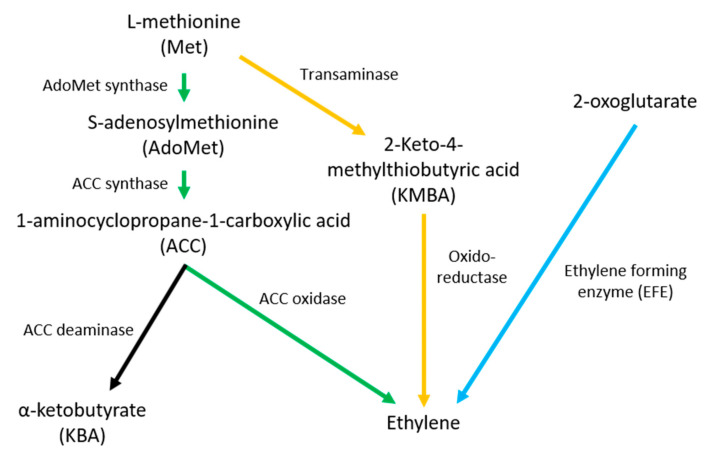
Ethylene biosynthetic pathways; the ACC pathway is shown in green, KMBA pathway in orange and oxoglutarate pathway in blue.

ET is the simplest olefin which acts in plants as a growth hormone inducing fruit ripening, flower senescence and leaf abscission [25]. Plants synthesize ET in a three-step reaction from methionine via S-adenosyl-methionine and the precursor 1-aminocyclopropane carboxylic acid (ACC) (Figure 1) [26]. Different microbes have been reported to be capable of ET production as well. While one of the microbial pathways also starts from methionine which is converted in a two-step reaction via 2-keto-4-methylthiobutyric acid catalyzed by a NADH:FeEDTA oxidoreductase [27], the other one uses 2-oxoglutarate as substrate which is directly converted to ET by the ethylene forming enzyme (EFE) [28,29,30]. 

The ET downstream signaling pathway induces ethylene response factors (ERFs) triggering the stress response. One possibility to alleviate stress is the degradation of the immediate precursor of ET, ACC, using an ACC-deaminase which has been described in plant growth promoting rhizobacteria (reviewed by Etesami and Maheshwari [31]). Also the plant pathogenic fungus *Fusarium graminearum* has a functional ACC deaminase in its genome, however, this gene does not have any impact on the virulence of the fungus on the highly susceptible wheat cultivar Apogee [32]. Plant growth promoting rhizobacteria (PGPR) have been reported to show antifungal activity against various plant pathogenic fungi of the genus *Fusarium*, *Colletotrichum*, *Aspergillus* and *Rhizoctonia* by producing plant growth promoting enzymes and hormones, cell wall degrading enzymes and antibiotics [33].

In *Capsicum annum* cv. Punjab Lal, a chili cultivar which shows an enhanced resistance towards *Colletotrichum truncatum* L., a micro RNA, Can-miRn37a, interacts with ERFs and represses downstream signaling. Overexpression of Can-miRn37a in a susceptible cultivar (Arka Lohit) results in resistance by preventing fungal colonization [34]. Expression of FaGAST2, a strawberry ripening related gene, is induced by ethephon, an intracellular generator of ethylene. The expression of that gene is enhanced by oxidative stress as well as infection by *Colletotrichum acutatum* while overexpression caused a delay in growth of strawberry plants [35]. Ethephon induces the expression of FaGAST2 upon infection as well as the delayed growth in overexpression lines. It remains to be investigated in how far overexpression of FaGAST2 has an impact on the levels of other plant hormones like auxin. 

Ethylene insensitivity has been described in *Arabidopsis*, wheat and barley to enhance resistance against *Fusarium graminearum* while ethylene overproducing lines exhibit increased susceptibility [36]. In contrast, ethylene insensitive lines of *Nicotiana tabacum* exhibit higher susceptibility upon inoculation with *Colletotrichum destructivum* compared to the wild type strain [37]. Since *F. graminearum* has been reported to produce ethylene on media with 20 mM methionine supplemented [32] reduced ethylene perception results in reduced stress upon *Fusarium* infection. While ethylene production has been documented in *Colletotrichum musae* [38] as well as *F. graminearum*, to our knowledge *C. destructivum* is not able to produce ethylene to increase virulence shedding light on the opposing effect of reduced ethylene sensitivity.

The rubber tree (*Hevea brasiliensis*) shows different symptoms upon infection with *Colletotrichum siamense* and *C. australisinense*. This diverged pattern was traced down to a different set of pathogenicity related genes [39]. Necrosis and ethylene-inducing peptide 1-like proteins (NLPs), which can be divided in different subgroups, are produced during infiltration of the extracellular space in dicots. The majority of the NLPs in *C. siamense* belong to subgroup II, which do not induce necroses in the host plants while ChNLP1 of *C. higginsianum* has been shown to induce necrosis in plants [40].

## 3. Abscisic Acid

**Figure 2:**
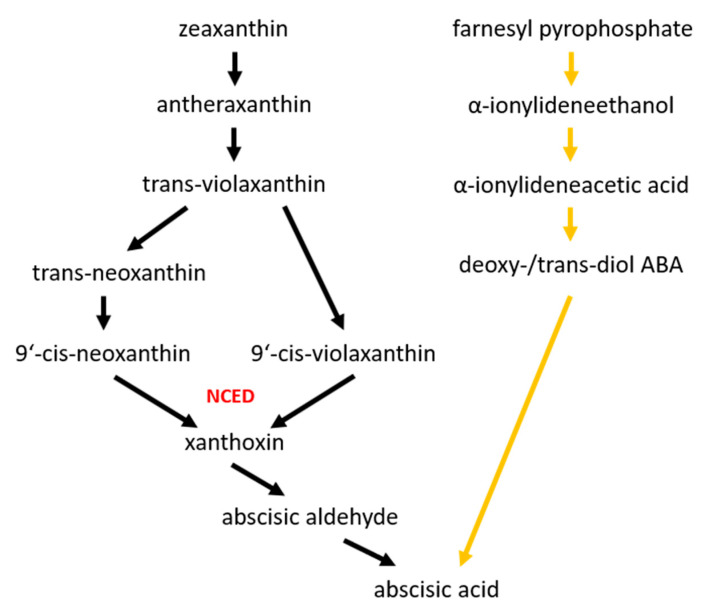
Abscisic acid biosynthetic pathways; the C_15_ pathway is indicated by orange arrows.

Abscisic acid, a sesquiterpenoid, acts as a plant signaling molecule mediating seed dormancy, bud growth and adaption to environmental stresses [41]. In plants, ABA is synthesized via the carotenoid biosynthetic pathway starting in the plastids. The nine-cis-epoxycarotenoid dioxygenase (NCED) catalyzes the rate limiting step, the cleavage of 9′-*cis*-neoxanthin or 9′-*cis*-violaxanthin. The resulting xanthoxin is converted to absicisic acid in the cytosol (Figure 2). Additionally, fungi also use a “direct pathway” via mevalonate where the intermediates contain no more than 15 carbon atoms [42]. The dynamics, signaling and functions of abscisic acid in plants have recently been reviewed by Chen et al. [43].

For several plant-pathogen systems, the ability of the pathogen to interfere with the host on plant hormonal level has been described [44]. Although several fungal species are not only able to synthesize ABA they are capable of sensing the hormone using specific receptors. In *Aspergillus nidulans* growth and metabolism can be regulated by abscisic acid. Already in the presence of 100 nM ABA spore germination as well as fungal growth are promoted [45]. The virulence of *Colletotrichum acutatum* in pepper fruits is enhanced in the presence of ABA which results in increased length of lesions. *Capsicum baccatum* cultivars which are generally resistant to anthracnose, showed symptoms of pepper anthracnose upon ABA administration indicating that exogenous ABA results in the suppression of defense mechanisms of pepper fruits against anthracnose [46]. The ABA receptor, PYR/PYL family, as well as brassinosteroid insensitive 1-associated receptor kinase 1, and ABA responsive element binding factor are lower expressed in the anthracnose resistant cultivar by the factors −25.2, −3.44, −2.73, −2.17, respectively, compared to the parent cultivar which shows a higher susceptibility [47] indicating the contribution of ABA to virulence. The infection promoting effect of ABA was also reported for the infection of rice by *Magnaporthe grisea* in combination with cold stress [48]. The ET and the ABA pathway have been demonstrated to be connected in *Arabidopsis* by one gene, ETHYLENE INSENSITIVE2 (EIN2) which is also called ENHANCED RESPONSE TO ABA3 (ERA3) [49]. ABA interacts antagonistically with the ET pathway indicating that altered ABA levels repress the ethylene induced defense response.

Tea plants produce volatile compounds to elicit defense in undamaged tissue and neighboring plants. (*E*)-Nerolidol triggers a mitogen-activated protein kinase, WRKY, which acts as transcription factor and is a key compound in the abscisic acid signaling pathway. It also induces an H_2_O_2_ burst and increased levels of jasmonic acid and enhanced abscisic acid signaling which can increase the antioxidant capacity upon stress [50]. However, ABA also enhances the activity of chitin deacetylase in submerged culture by the factor of 9.5 in *C. gloeosporioides* resulting in a lower degree of acetylation compared to a culture lacking ABA. This resulted in a change of the acetylation of chitin which was extracted from the cell wall [51]. Chitin deacetylases (CDAs) from several *Colletotrichum* species have previously been expressed and characterized [52,53,54]. CDAs in fungi catalyze the deacetylation of chitin which leads to the formation of chitosan. This deacetylation is necessary for several fungal pathogens to increase virulence. Especially soil borne fungal pathogens have been reported to use this deacetylation as a major virulence strategy [55].

On the other side, chitinases in plants are supposed to degrade chitin, a major component of the fungal cell wall. Since most antifungal peptides are highly basic, the positive charges of chitinases might facilitate electrostatic interactions with the negatively charged phospholipids on the fungal cell surface. However, several plant pathogenic fungi secrete proteases acting against antifungal plant chitinases (reviewed by [56]). Knock down of CaChiIII7 in pepper plants resulted not only in hypersensitivity to *C. acutatum* but also in attenuated defense response genes CaPR1, CaPR5 and SAR8.2 [57]. 

Postharvest treatment of mangoes with chitosan revealed downregulated abscisic acid and jasmonic acid levels in the peels, concomitant with a significantly extended shelf life. An infection experiment with *C. gloeosporioides* showed that anthracnose lesions were significantly smaller on fruits treated with chitosan compared to ones treated with acetic acid and water [58].

## 4. Auxin

Indole-3-acetic acid (IAA) which is also known as auxin acts as a growth hormone mediating apical growth and root morphology and gravitropism. Several tryptophan (TRP) dependent and TRP independent pathways have already been described in plants, algae, bacteria and fungi [59]. Indole-3-acetic acid production from TRP has been reported in *C. gloeosporioides* f. sp. aeschynomene in 1998. Beside auxin also tryptophol (TOL) and indole-3-acetamide (IAM) were detected in this study indicating that the IAM pathway is used [60]. Another study two years later showed as well that *Colletotrichum* sp. is capable of auxin production which was confirmed by NMR analysis [61]. Subsequent research revealed that *C. acutatum* is able to produce auxin from tryptophan. Beside IAA, the intermediates indole-3-acetaldehyde (IAAld), IAM as well as indole-3-pyruvic acid (IPA) were detected suggesting that different auxin biosynthetic pathways are used [62]. Also, *C. fructicola* which was isolated from coffee plants has been reported to produce auxin in vitro from TRP using the IAM pathway [63]. 

**Figure 3:**
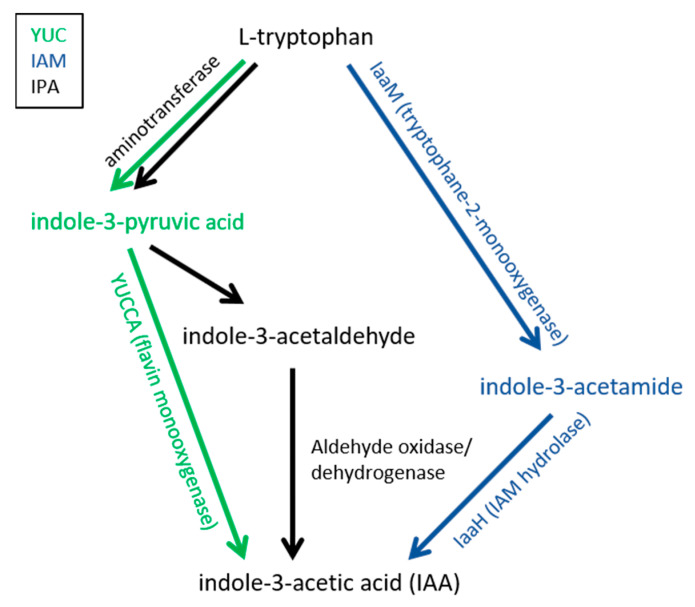
Tryptophan derived auxin biosynthetic pathway in plants (YUC (green)) and proposed pathways in *Colletotrichum* spp. (IAM (violet), IPA (black)).

Based on the previously reported results the proposed auxin biosynthetic pathways in *Colletotrichum* emanate from tryptophan (Figure 3). While in plants the yucca pathway via IPA which is directly converted to auxin is used, *Colletotrichum* synthesizes IAA either using the IAM pathway (blue) or the IPA pathway via IPA and IAAld (black). 

IAA is often involved in plant-pathogen interaction, but it is also used by fungi to increase virulence and is therefore rather involved in plant disease susceptibility (reviewed by Chanclud and Morel [64]). Upon increasing auxin concentrations, Aux/IAA transcriptional repressors are removed from auxin response factors (ARF). Further, TIR1/AFB can bind to Aux/IAA transcriptional repressors inducing polyubiquitylation which further leads to proteasomal degradation. Negative feedback loops are triggered by the induced auxin responsive genes to which Aux/IAAs and the GH3 family are counted [65]. *C. gloeosporioides* f. sp. aeschynomene produces IAA in axenic culture using the IAM pathway and auxin is also formed at an early stage of infection indicating contribution to virulence [66]. This has been shown as well in *Fusarium* pathogenic to Orobanche. Introducing two genes of the indole-3 acetamide pathway in *F. oxysporum* and *F. arthosporioides* resulted in significantly higher auxin production concomitant with hypervirulence [67] supporting that fungal auxin production contributes to virulence.

A transcriptomic analysis of strawberry leaves inoculated with *C. fructicola* revealed that 24 h post inoculation JA and IAA levels were higher compared to the mock treatment while SA and ABA peaked after 48 h, however, the changes were not significant at any timepoint [68]. Another study investigating the interaction between *Colletotrichum camilliae* and tea plants (Longjing 43) demonstrated that the precursors and the intermediate products of JA and IAA biosynthesis significantly increased during the interaction, in particular when the symptoms became apparent [69]. Analysis of selected microRNAs (miRNAs) of *Camellia sinensis* upon *C. gloeosporioides* infection revealed five miRNAs which are involved in the regulation of the auxin signaling pathway. Phenylalanine ammonia lyase (PAL) and cinnamoyl-CoA reductase (CCR) were identified as target genes as well [70]. PAL deaminates L-phenylalanine into *trans*-cinnamic acid which can be further converted into *p*-coumaric acid by cinnamate-4-hydroxylase (C4H). It has been described that auxin-regulated plant growth is fine-tuned by early steps in phenylpropanoid biosynthesis in terms of reduced PAL expression, while loss of C4H increases the strength of the auxin response [71]. 

## 5. Salicylic Acid

**Figure 4:**
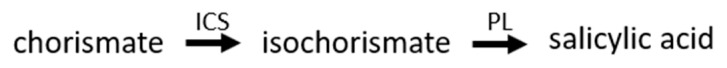
Salicylic acid biosynthesis pathway.

Salicylic acid (SA) plays an essential role in the activation and regulation of responses to biotic and abiotic stresses. The biosynthesis of SA emanates from the shikimate pathway with the conversion of chorismate to isochorismate (IC) by isochorismate synthase (ICS). IC is further cleaved by pyruvate lyase (PL) releasing pyruvate and SA (Figure 4) [72]. 

In contrast to auxin, *Colletotrichum* spp. have not been reported to be capable of producing SA. SA is involved in the resistance of tea plants to anthracnose infection. The total amount of SA (bound and free SA) is approximately twice as high in anthracnose infected tea leaves compared to healthy leaves [73]. Several studies describing the SA levels of different host plants upon *Colletotrichum* infection have been published.

**Figure 5:**
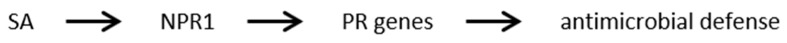
Salicylic acid signal transduction.

SA is required for induction of the systemic acquired resistance (SAR) through NPR1-regulated expression of pathogenesis related (PR) genes (Figure 5) [74]. Methyl salicylate (MeSA) acts over a long distance in *Arabidopsis* and tobacco. For activation, cleavage of MeSA by a MeSA esterase is necessary to release SA [75]. In strawberry, transcripts associated with systemic acquired resistance (SAR) and SA-mediated signaling (like FaMeSA1, a methyl salicylate esterase) pathways increase upon *C. acutatum* infection [76]. 

The Gretchen Hagen 3 (GH3) gene family which belongs to the early auxin responsive families is ubiquitous in the plant kingdom. It is involved in modulation of hormone homeostasis and adaption to different stresses. GH3, an auxin amido synthetase, has been reported to alter the ratio of IAA and phenylacetic acid (PAA), which is also known as a plant growth substance, in *Arabidopsis* by catalyzing the formation of IAA-Asp, IAA-Glu, PAA-Asp and PAA-Glu [77]. Crossing of SA-accumulating *Arabidopsis thaliana* lines with IAA-overproducing mutants revealed that most of the phenotypes associated with IAA-overproduction (long hypocotyls, epinastic cotyledons, narrow rosette leaves during the adult stage and increased apical dominance) are suppressed by SA accumulation indicating a coupled regulation [78]. GH3.5 acts as positive modulator of SA signaling and plays an important role in auxin-elicited susceptibility as well. On one side GH3.5 positively regulates IAA accumulation during pathogen infection but on the other side it shows an adenylation activity on SA [79]. Expression of auxin responsive GH3-like protein is highly elevated in *Citrus madurensis* flowers upon *C. acutatum* infection while IAA amino acid hydrolase and a putative growth regulator protein do not seem to be affected [80]. It was documented that strawberries with a higher resistance towards *Colletotrichum gloeosporioides* show higher basal SA levels which rapidly increase upon infection. Besides, SA directly inhibited the germination of *C. gloeosporioides* conidia as well. Also exogenous application of SA four days before infection resulted in reduced symptom development [81]. SA affects plant growth under stress by stomata regulation, water relations, nutrient uptake as well as photosynthesis. Exogenous application of SA enhances resistance towards various pathogens in monocot and dicot plants (reviewed by [82]).

Furthermore, exogenous application of SA leads to enhanced resistance of cassava to *C. gloeosporioides* due to induction of syntaxin gene expression. Syntaxins generally contribute to mediate vesicle fusion in trafficking fusion by specifically forming as ternary soluble N-ethylmaleimide-sensitive factor attachment protein receptors (SNAREs) complex with the purpose to transport defense compounds to the site of microbe infection between plant cell wall and plasma membrane [83]. Anthracnose severity in cassava can be reduced by 33.3% upon SA administration under greenhouse conditions under which β-1,3-glucanase and chitinase enzyme activities were significantly higher after 24 h post inoculation. However, the activity decreased 48 h after challenging the plants with *Colletotrichum gloeosporioides* [84]. SA levels in different host plants upon *Colletotrichum* infection are shown in Table 1.

## 6. Jasmonic Acid

**Figure 6:**
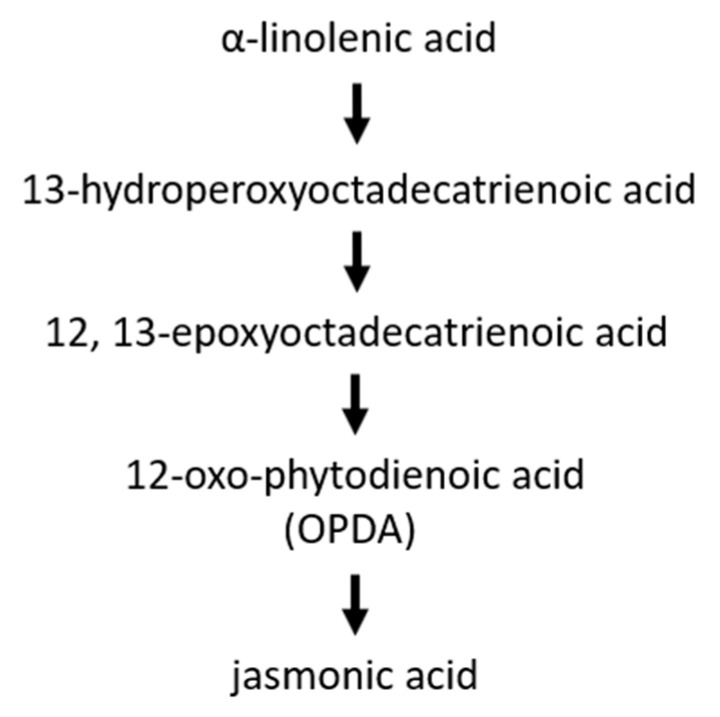
Jasmonic acid biosynthesis.

Jasmonic acid (JA) and intermediates occur in higher plants, some lower plants as well as in some prokaryotes. The biosynthesis is a route of oxidative reactions of lipid-derived fatty acids with 12-oxo-phytodienoic acid (OPDA) as intermediate (Figure 6; reviewed in [89]).

Activation of JA signaling is essential for plant resistance against necrotrophic pathogens [90]. While tea plants treated with laminarin, a β-glucan, show rapid accumulation of CsMAPK and CsWRKY3 as well as accumulation of high levels of SA but not JA, induction of these two genes by (*E*)-nerolidol, a volatile signal compound, results in increased levels of JA, JA-Ile, H_2_O_2_ and ABA but not SA. Disease symptoms induced by *C. fructicola* are suppressed by JA application indicating involvement of JA in the defense of tea plants against the pathogen [50]. A qPCR study where two coffee varieties were inoculated with *C. kahawae* revealed that JA is earlier and stronger induced in the resistant variety indicating its role in successful activation of defense responses and inhibition of fungal growth [91] which is associated with the hypersensitive-like host cell death as well as the early accumulation of phenolic compounds in the cell walls and in the cytoplasmatic contents [92,93]. *C. gloeosporioides* shows enhanced virulence on tea leaves under elevated CO_2_ levels which also reduced the caffeine content in the leaves while exogenous caffeine application reduced size of the lesions induced by the fungus. Upon caffeine administration lipoxygenase (LOX) activity and its gene expression is elevated regardless of the CO_2_ level. However, in presence of fungal infection the JA content is only increased under elevated CO_2_ indicating the presence of a JA-independent LOX pathway in tea plants [94]. JA is also involved in the susceptibility of maize plants towards anthracnose caused by *C. graminicola*. Inoculation of a JA deficient double mutant, opr7opr8, with *C. graminicola* showed elevated resistance. Analysis of the hormones revealed enhanced levels of SA while JA levels were strongly diminished [85]. opr7opr8 are crucial for JA biosynthesis and hence play an important role in the development of plants, however, these defects can be rescued by exogenous application of JA. JA deficient mutants also show a longer lifespan of the first and the second leaf. While ET levels are comparable between the wild type and the opr7opr8 mutants in the first leaf after 12 days, ABA levels are significantly lower in the mutant leaves which is expected due to the delayed leaf senescence [95]. Jasmonic acid plays a role in resistance towards insects and necrotrophic fungal pathogens. However, several pathogens evolved mechanisms to hijack the JA pathway. For example, *Pseudomonas syringae* pv. Tomato secretes virulence effector protein as well as a polyketide phytotoxin to interfere with and evade the plant defense system. Also, beneficial microbes are able to hijack JA homeostasis to establish symbiotic interactions (reviewed by [96]).

## 7. Brassinosteroids

Brassinosteroids (BRs) belong to the class of steroid plant hormones. Free BRs either contain 27, 28 or 29 carbon atoms within their skeletal structure. They are either synthesized via the mevalonate or the non-mevalonate pathway which has recently been reviewed by Bajguz et al. [97]. Signaling and signal transduction have recently been reviewed as well [98]. BR signaling briefly summarized: In the absence of BRs BRI1 and BAK1 which are plasma membrane localized receptors, are inhibited by several factors including BKI1 and BIR2. BIN2 kinase phosphorylates BES1 and BZR1 transcription factors acting as a negative regulator. This results in a higher expression of BR repressed genes while BR-induced genes are hardly expressed. On the other side, in the presence of BRs, the hormone binds to the BRI1 receptor and the BAK1 co-receptor initiating the signaling cascade. This leads to the dissociation of BKI1 and BAK1 from the receptor which are further activated by phosphorylation. Next, BSKs/CDGs become phosphorylated activating BSU1 phosphatase which inhibits BIN2. After dephosphorylation of BES1 and BZR1 by PP2A, BES1 and BZR1 can interact with transcription factors and cofactors promoting BR-induced gene expression and inhibit the expression of BR-repressed genes.

BRs also play a role in the regulation of other plant hormones. Auxin response factors (ARFs) are transcriptionally regulated by BRs in a transcriptional feedback loop [99]. BIN2 mediated phosphorylation of ARF2 has been demonstrated to reduce ARF2 DNA binding and repression activities [100]. The crosstalk between gibberellins (GA) and BRs is mainly achieved through GA induced degradation of DELLA since active GAs are bound to the GIBBERELLIN INSENSITIVE DWARF1 (GID1) receptor. As a result, GID1 binds to the N-terminal region of DELLA proteins which induces their degradation via the ubiquitin-proteasome pathway [101]. BRs are also involved in plant-pathogen interactions regardless of whether the interactions are biotrophic, hemibiotrophic or necrotrophic (reviewed by [102,103]).

Exogenously applied BRs give plants resistance or tolerance to different abiotic stresses but also induce protection against different pathogens. A study where strawberry plants were treated with 24-epibrassinolide (EP24) and a brassinosteroid spirostanic analogue DI-31 (BB16), the resistance towards *C. acutatum* was enhanced concomitant with increased production of H_2_O_2_, O_2_^−^, NO, calcium oxalate crystals as well as higher callose and lignin deposition [104]. An RNA-seq approach with red mango fruits which were inoculated with *C. gloeosporioides* revealed not only upregulated ethylene related gene expression but also enhanced expression of genes belonging to the phenylpropanoid and brassinosteroid pathways [105]. BRs have also been described to induce disease resistance in *Nicotiana tabacum* and *Oryza sativa* [106]. A recently delineated link between brassinosteroid and JA signaling suggests that OsGSK2, a key suppressor of BR signaling, also enhances on one side antiviral defense but also activates JA signaling [107].

## 8. Synopsis

**Figure 7:**
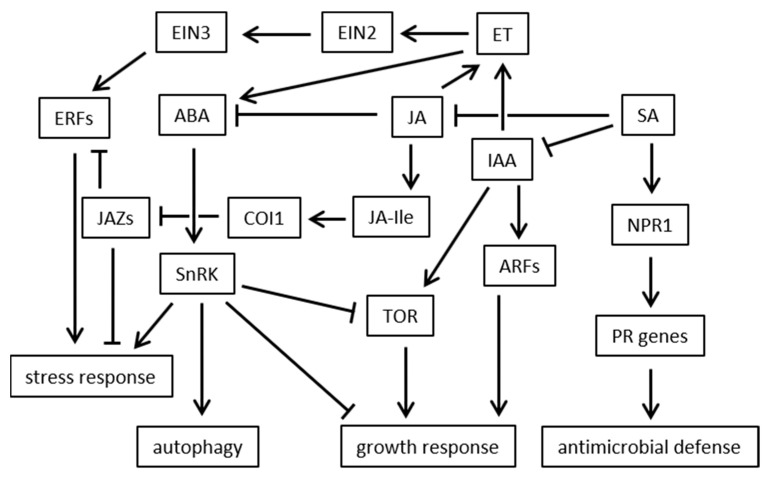
Simplified model of the contribution of different plant hormones to stress response; SA reduces the formation of IAA and induces the expression of non-expressor of pathogenesis related gene 1 (NPR1). Localization of the NPR monomer in the nucleus activates TGA transcription factors (TFs) which can bind pathogenesis related (PR) gene promoters and activate transcription of defense genes. JA is induced upon biotic and abiotic stresses. It is converted to the biologically active form JA-Ile which is perceived by its receptor COI1. COI1 triggers the degradation of JAZ repressors resulting in the release of downstream transcription factors and further induction of JA-responsive genes. JA induces ethylene formation which activates ethylene insensitive 2 (EIN2), a transmembrane protein, which further induces the transcription factor EIN3 leading to expression of ethylene response factors (ERFs) triggering the stress response. ABA induces stress response and autophagy via SNF1-related protein kinase (SnRK) which on the other side inhibits the growth response and the target of rapamycin (TOR) but promotes stress response and autophagy. Auxin leads to the activation of auxin response factors (ARFs) as well as TOR which both trigger growth response.

Plant hormones play a crucial role in plant-microbe interaction regardless whether a symbiosis is formed, a pathogen interferes with plant hormone homeostasis during infection or in the defense of the plant triggering expression of stress responsive genes. Several *Colletotrichum species* have been described to be capable of auxin production, however, only the metabolic intermediates have been described [61,62,63,80]. Understanding the contribution of auxin to virulence during *Colletotrichum* infection may open new opportunities for resistance breeding. Since auxin acts as growth hormone it is supposedly not contributing to stress tolerance but rather weakens the stress response of the plant. A simplified model of the contribution of different plant hormones to stress response is shown in Figure 7.

Many symbionts and plant pathogens have evolved the ability to interfere with plant hormone homeostasis [63,108,109,110,111]. However, the literature dealing with plant—*Colletotrichum* interaction on plant hormonal level is limited, especially the biosynthetic pathways of plant hormones in *Colletotrichum* and their perception. There are still many open questions from the plant hormonal perspective. In how far auxin produced by *Colletotrichum* boosts virulence remains to be investigated. A more detailed description of the role of auxin, ethylene, abscisic acid and other plant hormones during *Colletotrichum* infection may show opportunities for directed plant breeding and enhance resistance this way.

## Figures and Tables

**Table 1:** Salicylic acid levels in different plants upon Colletotrichum infection.

Host Plant	Cultivar/Strain	Tissue	Pathogen	SA-Levels	Time Point	Reference
Maize	W438	Leaf	*C. graminicola*	~100 pmol/g FW	1 dpi	[85]
	lox10-3			~100 pmol/g FW		
	B73	Leaf		<100 pmol/g FW		
	opr7.5 opr8.2			>200 pmol/g FW		
Strawberry	Jiuxiang	Leaf	*C. fructicula*	0.36 ng/mg FW	1 hpi	[86]
	Benihoppe			0.38 ng/mg FW		
Arabidopsis	Col-0	Leaf	*C. higginsianum*	~90 µg·m^−2^	2 dpi	[87]
	sweet11			~110 µg·m^−2^		
	sweet12			~140 µg·m^−2^		
	sweet11/12			~190 µg·m^−2^		
Cucumber	*Cucumis sativus*	Cotyledon inoculated:	*C. lagenarium*			[88]
		Roots		2 µg/g	6 dpi	
		Leaf		0.6 µg/g		
		Hypocotyl		2 µg/g		
		First leaf inoculated:				
		Roots		>2.5 µg/g	7 dpi	
		Leaf		>3.0 µg/g		
		Hypocotyl		~3.0 µg/g		
Tea plants	Longjing 43 and Zhenong 139	Healthy leaves	*Colletotrichum* spp.	~8 µg/g FW	Collected in July	[73]
		Infected leaves		~13 µg/g FW		
Strawberry	Camarosa	Control leaf	*C. acutatum*	74.42 ng/g DW	3 dpi	[76]
		Infected leaf		202.21 ng/g DW		
		Control leaf		52.33 ng/g DW	5 dpi	
		Infected leaf		354.77 ng/g DW		
